# Social buffering by siblings in childhood and adolescence

**DOI:** 10.1016/j.psyneuen.2025.107580

**Published:** 2025-08-19

**Authors:** Deborah Han, Natalie A. Thwaites, Julia Olson, Kenia M. Rivera, Julia Dmitrieva, Jenalee R. Doom

**Affiliations:** a Department of Psychology, University of Denver, United States; b Graduate School of Professional Psychology, University of Denver, United States

**Keywords:** Social buffering, Siblings, Cortisol, Childhood, Adolescence, Depressive symptoms

## Abstract

Although positive sibling relationships are associated with better developmental outcomes for children and adolescents, it is unknown whether siblings may be effective social buffers of physiological stress. In addition, there is little research on whether depressive symptoms may moderate the effectiveness of social buffering. The current study examined (1) whether siblings, compared to strangers, buffered the cortisol response to a social evaluative stressor in children and adolescents, (2) whether depressive symptoms moderated this effect, and (3) whether self-reported sibling relationship quality influenced sibling buffering effectiveness. A total of 72 children (9–11 years) and 65 adolescents (15–17 years) were randomly assigned to complete the Trier Social Stress Test-Online with a same-age or older sibling versus a stranger. Participants provided saliva samples to measure cortisol reactivity and recovery across the session and reported on their depressive symptoms and sibling relationship quality. The sibling versus stranger buffering effect varied based on participants’ developmental stage (child vs. adolescent) and level of depressive symptoms. Specifically, adolescents with high depressive symptoms who prepared with a sibling showed lower cortisol reactivity than those who prepared with a stranger (*b* = 0.70, *SE* = 0.32, *p* < .05). In contrast, there were no significant differences by condition for children or adolescents with low depressive symptoms. For individuals who prepared with a sibling (*n* = 69), higher alienation predicted impaired cortisol recovery post-stressor (*b* = 0.32, *SE* = 0.18, *p* < .05). These findings are in line with research on normative development of sibling relationships and cortisol activity in the context of depression from childhood to adolescence.

## 1. Introduction

One of the most well-replicated findings in psychology is the association between positive social relationships and better mental and physical health (Cohen, 2004; Holt-Lunstad, 2018; Santini et al., 2015). While social relationships can affect health through several pathways, social buffering is one pathway by which relationships acutely affect stress physiology, and potentially long-term health. Social buffering is a phenomenon where the presence of a social partner reduces an individual’s physiological response to an acute stressor. This phenomenon has been studied in many species and across multiple physiological systems, including the hypothalamic-pituitary-adrenal (HPA) axis, which is commonly measured via activity of the hormone cortisol (Gunnar, 2017; Hostinar et al., 2014).

Much of the evidence for social buffering of the HPA axis in humans comes from experimental studies that manipulate the provision of social support before, during, or after a lab-induced stressor (e.g., Trier-Social Stress Test [TSST]). In this paradigm, the presence or assistance of a social partner is often compared to a control condition, such as no support or support from a stranger. Numerous studies have used the TSST to test the effectiveness of parents and friends as social buffers of the cortisol stress response for children and adolescents (Gunnar, 2017). Across these studies, effective buffering has been characterized by diminished cortisol reactivity in response to the stressor (Hostinar et al., 2015a, 2015b; Doom et al., 2015, 2017; Parenteau et al., 2020; Filetti et al., 2025), as well as quicker recovery, or return to baseline levels of cortisol, post-stressor (Seltzer et al., 2010; Doom et al., 2015; Calhoun et al., 2014).

One potential support figure yet to be explored in social buffering research is siblings. Given the unique role that siblings play in shaping development (McHale et al., 2012), there is reason to believe that they could serve as effective buffers of physiological stress responses. For example, positive sibling relationships are associated with better psychosocial outcomes in children and adolescents (Branje et al., 2004; Buist et al., 2013; Yeh and Lempers, 2004), even after accounting for parent and peer relationships (Harper et al., 2016; Kim et al., 2007). Furthermore, sibling support has been shown to protect against the negative effects of parental divorce (Kempton et al., 1991), stressful life events (Gass et al., 2007), and social problems (Fry et al., 2021) on internalizing and externalizing behaviors. These studies, however, do not establish causality and only provide correlational evidence of stress-protective effects based on self-report measures. These studies also lack measures of physiological responses to stress. The current study is the first to use an experimental paradigm to investigate whether siblings can buffer the physiological stress response to a lab-induced stressor in children and adolescents. In particular, we tested whether preparing for a social evaluation task with a sibling is associated with a lower cortisol response to the task and quicker return to baseline than when preparing with a stranger.

There is growing evidence for individual differences in social buffering effectiveness based on characteristics of the person receiving or giving support (Hostinar et al., 2015b; Parenteau et al., 2020). One factor that may be particularly salient for social buffering in children and adolescents is one’s level of depressive symptoms. Children and adolescents with major depressive disorder or at high risk for depression tend to exhibit dysregulated HPA responses to stress, including patterns of cortisol hyperactivity (i.e., increased reactivity and prolonged recovery; Rao et al., 2008; Stewart et al., 2013) or hypoactivity (i.e., blunted reactivity; Luby et al., 2003, 2004). While most research focuses on cortisol reactivity as preceding the development or onset of depression (Guerry and Hastings, 2011), depressive symptoms may also affect concurrent cortisol activity, including sensitivity to social regulation of cortisol responses. This idea stems from research on depression’s association with social dysfunction, including more interpersonal difficulties and heightened sensitivity to negative events (Kupferberg, Hasler, 2023; Verboom et al., 2014; Steger and Kashdan, 2009). It is therefore possible that individuals with depression or at high risk for depression may not benefit from social buffering as much as those with low depressive symptoms. This possibility has rarely been tested in the social buffering literature.

In addition to whether siblings can buffer physiological stress, it is important to understand what aspects of sibling relationships may influence their buffering potential. Unlike friendships, sibling relationships do not dissolve if there are issues with the quality of the relationship, so the quality of relationships may be more variable than friendships. Previous research indicates that both positive and negative dimensions of sibling relationship quality, such as intimacy versus conflict, uniquely contribute to development (Buist et al., 2013). A previous study by Calhoun et al. (2014) examined whether positive and negative friendship qualities differentially affect buffering of the cortisol stress response by friends in adolescents. While there were no main effects of positive friendship qualities on participants’ cortisol reactivity or recovery slopes, higher levels of negative friendship qualities were associated with impaired cortisol recovery (i.e., slower return to baseline) following the stressor task (Calhoun et al., 2014). The current study similarly investigated whether positive and negative sibling relationship qualities differentially predict sibling buffering effectiveness.

### 1.1. Current study

To summarize, the present study tested whether preparing for a social evaluation task with a sibling compared to a stranger buffered the cortisol stress response to the task for children and adolescents. To get a better understanding of HPA responses to stress, we examined both reactivity and recovery components of the stress response. We hypothesized that both children and adolescents who prepared for the social evaluation task with a sibling would show a more adaptive cortisol stress response to sibling buffering (i.e., lower cortisol reactivity and quicker recovery to baseline) than those who prepared with a stranger. Based on previous research studies that have demonstrated lower effectiveness of parental and friend buffering for adolescents (Doom et al., 2015, 2017; Hostinar et al., 2015a), we hypothesized that the buffering effect of siblings would be stronger for children compared to adolescents. We conducted exploratory analyses to test whether the effect of condition (sibling versus stranger) differed by participants’ levels of depressive symptoms. We hypothesized that high levels of depressive symptoms would reduce the effectiveness of sibling social buffering for both children and adolescents. Finally, we conducted exploratory analyses of whether the sibling buffering effect (for those who prepared with a sibling) differed by self-reported sibling relationship quality. We hypothesized that positive qualities (communication and trust) would be associated with more adaptive cortisol responses to sibling buffering (i.e., lower reactivity and quicker recovery) while negative qualities (alienation) would be associated with more impaired cortisol responses (i.e., hyper-elevated reactivity and slower recovery).

## 2. Material and methods

### 2.1. Participants

A total of 79 children (9–11 years)^1^ and 71 adolescents (15–17 years) were enrolled as primary participants for the study. Each primary participant participated with a same-age (i.e., twin) or older sibling, and a parent/guardian. As a result, each family that participated in the study had three total family members involved in study procedures. We restricted the age of the sibling to the same age or up to four years older than the primary participant as the type of support that individuals receive from their siblings and the extent to which they benefit from sibling support may differ based on whether the sibling is older versus younger (Fry et al., 2021; Tucker et al., 2001). All study procedures were virtual, though participants were asked to collect saliva samples at home and send them to the laboratory by mail.

Families were recruited from a psychology department’s participant pool of families interested in research, public flyers, social media postings, snowball sampling (i.e., referrals from families who participated in the study), and via older siblings in psychology classes at the university who participated in the study for course credit. All parents completed an initial screening call to determine eligibility before scheduling a session. Exclusion criteria for the primary participant included: (1) current foster care placement, (2) severe learning disability that would interfere with their ability to complete the study session, (3) regular use of steroid medications (e.g., daily inhaler use for asthma), (4) diagnosis of a disorder known to affect cortisol levels (e.g., Cushing’s or Addison’s disease), and (5) aged 9–11 years and past the mid-point of puberty or aged 15–17 years and not past the mid-point in puberty (assessed via The Pubertal Development Scale, as in previous social buffering studies; Carskadon and Acebo, 1993; Doom et al., 2015; Petersen et al., 1988). Of the 150 child/adolescent participants who completed the study session, a total of 13 participants (8.67 %) were excluded from further analyses for the following reasons: family did not return saliva samples (*n* = 8), incomplete child survey (*n* = 2), participant reported recent use of steroids which was reflected in extremely high cortisol values (*n* = 2), and undetectable cortisol values for most samples (*n* = 1).

Thus, the final sample for this study included the families of 72 children (*M_age_* = 9.91, *SD* = 0.64; 48.61 % female) and 65 adolescents (15–17 years, *M_age_* = 15.75, *SD* = 0.75; 49.23 % female). Demographic and descriptive data for the study sample are presented in Supplemental Table 1. The majority of primary participants (74.45 %) identified as non-Hispanic/Latinx White, 13.87 % identified as Hispanic/Latinx, 8.76 % identified as Asian, and 2.92 % identified as Other. Sibling age ranged from 10 to 20 years (*M_age_* = 15.06, *SD* = 3.36) with a mean age difference of 2.43 years (*SD* = 0.95) from the primary participant. Most sibling pairs (91.24 %) were full biological siblings (i.e., shared the same biological mother and father) and almost half of the sibling pairs (48.91 %) identified as the same gender. Regarding socioeconomic status, 3.65 % of parents reported family incomes of $50,000 or less, 8.03 % reported incomes of $50,001–$100,000, 25.55 % reported incomes of $100,001–$150,000, 27.01 % reported incomes of $150,001–$200,000, 32.12 % reported incomes of $200,001 or more, and five parents (3.64 %) did not report income. Parental education ranged from completion of high school/GED to graduate degree (e.g., master’s or beyond), though most parents (86.13 %) reported having a bachelor’s degree or higher.

### 2.2. Procedure

**Fig. 1.**
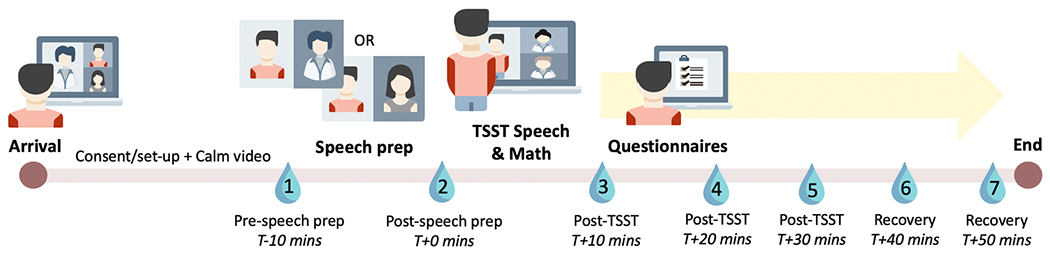
Timeline of study session and saliva samples. Notes: Times shown here are the saliva sample collection points relative to start of TSST speech. The primary participant provided saliva sample #1 at 32.18 min (*SD* = 5.61) on average after the start of the session. All siblings joined the Zoom call with primary participants to begin the session and were present during the consent/set-up portion. Siblings were then separated from the primary participant before the calming video and for the remainder of the session, unless they were the helper for speech prep. Note that the only difference between the two conditions (sibling versus stranger) was during the 5-min speech prep period.

Participants completed one 2-hour virtual session via Zoom that was scheduled to begin between 2 and 4 PM in the primary participant’s time zone to control for diurnal cortisol variation. We describe in depth below the session timeline (See Fig. 1) and procedures, which were adapted from the Trier Social Stress Test-Online (TSST-OL) protocol by Gunnar et al. (2021). Because this original protocol did not include a social buffering component, modifications were made as described below for the current study. Data collection began in November 2021 and ended in July 2023. Months since the onset of the COVID-19 pandemic (March 11, 2020, as declared by the World Health Organization) to the session date ranged from 20 to 39 months (*M* = 32.68, *SD* = 4.91).

#### 2.2.1. Pre-session

After recruitment and scheduling, a saliva collection kit was sent to the family’s home, and researchers confirmed receipt of the kit before the session date. Primary participants were instructed to avoid caffeine for at least 30 min before the session and to avoid eating or drinking anything except water for at least 15 min before the session. Ahead of the session, families were asked to use one device with at least an 11” screen for the primary participant to join from (e.g., laptop, chrome book) and another device for the sibling to join from (e.g., iPad, tablet, phone). Primary participants and siblings joined the Zoom meeting from separate rooms if they were living in the same house. Parents were required to complete the consent form before or at the beginning of the session and asked to complete their questionnaire by the end of the session day. Primary participants were randomly assigned to sibling versus stranger condition before each session but were not told about their assignment until the speech preparation period during the session.

#### 2.2.2. Consent and set-up

Upon arrival, the experimenter completed consent/assent with all participants and then walked the participants through study set-up. The experimenter reviewed Zoom settings with the participant and confirmed: 1) the internet connection was sufficient, 2) they were in a private area with a closed door, 3) there were no pets in the room (except those in tanks that could not be easily moved), 4) all cellphones were turned off or on “do not disturb,” and 5) they could stand and back up enough to be seen from their head to hands while maintaining clear visuals and audio. The experimenter then demonstrated how to collect the saliva samples.

#### 2.2.3. Calming video

Parents were asked to leave the room after consent and set-up, and the experimenter moved the primary participant and their sibling to separate Zoom breakout rooms. Siblings filled out their questionnaires during this time. The experimenter joined the primary participant’s breakout room and shared their screen to play a calming children’s movie for 10 min. Then the primary participant provided their first saliva sample (sample #1), which was on average 32.18 min (*SD* = 5.61) after the start of the session.

#### 2.2.4. Speech preparation

After sample collection, the experimenter announced the random assignment of sibling or stranger for speech preparation to the primary participant. The primary participant remained in their breakout room (stranger condition) or were moved to their sibling’s breakout room (sibling condition). Participants were instructed to prepare a speech introducing themselves to a new classroom of peers and were told that their video would be recorded so that it could be judged compared to others later. They were given 5 min to prepare and were told that the experimenter (stranger condition) or their sibling (sibling condition) was there to help them. Participants were allowed to use a pencil and paper or an electronic document on their computer to prepare. The experimenter either kept their video on and made themselves available (stranger condition) or turned their video and audio off (sibling condition) throughout the duration of speech preparation. After speech preparation in both conditions, the primary participant was moved to a breakout room with the experimenter and two trained research assistants joining as “judges”. Although the speech preparation period itself only lasted 5 min, the time duration between sample #1 and TSST start was closer to 10 min given technical steps of moving between breakout rooms and giving instructions.

#### 2.2.5. TSST stress paradigm

Once the primary participant joined the judges’ breakout room, they were prompted to provide saliva sample #2. Afterwards, both judges started recording and Judge 1 instructed the participant to stand up and back away until they were visible from their head to hands. The primary participant then completed the speech and math portions of the TSST-OL for 5 min each (Gunnar et al., 2021). After TSST completion, the primary participant provided saliva sample #3 and was moved back to the experimenter’s room.

#### 2.2.6. Post-TSST

Once reunited with the experimenter, the primary participant was sent a link to their questionnaire in the chat. While completing the questionnaire, the experimenter prompted the participant to take saliva sample #4 at the 20-min mark after TSST start (beginning of speech delivery) and samples #5–7 every 10 min thereafter. Once all samples were collected, participants were debriefed about the nature of the TSST and told that their video would not be shown to anyone outside of the researchers. Parents were asked to complete their questionnaires and mail saliva samples back to the lab.

### 2.3. Salivary cortisol

Seven saliva samples were collected throughout the session via passive drool (see Fig. 1 for timing of samples relative to stressor onset). After the session, parents were instructed to ship the saliva samples to the researchers within 48 h using a pre-labeled and pre-paid package. Once received, samples were stored in a laboratory freezer at −80 degrees Celsius until they were shipped to the University of Trier for assay.

Samples were assayed for cortisol concentrations in nmol/L using a time-resolved fluorescence immunoassay. The intra-assay coefficients of variation (CV) were between 4.00 % and 6.70 %. The inter-assay CVs were between 7.10 % and 9.00 %. All seven samples for each participant were included in the same assay batch to avoid between-batch variation, and batches were balanced by age group and condition. Samples were assayed in duplicate for cortisol and average values were used in analyses whenever possible, while a single value was used if there was not enough saliva for duplicate assays (*n* = 17 samples). All cortisol values were examined for outliers, and values that were three standard deviations from the mean were winsorized to the highest value within three standard deviations from the mean (*n* = 24; 2.54 % of total samples). Consistent with previous literature on cortisol, all values were then natural log-transformed to address positive skew.

### 2.4. Measures

#### 2.4.1. Daily diaries

Primary participants and their parents completed daily diaries to report on variables relevant to cortisol collection, including waketime and medication usage of the primary participant. While participant self-reports were prioritized, parent reports were used when participant reports were missing or insufficient. Time since wake relative to session start time was calculated in hours. After excluding individuals who took medications known to affect cortisol levels (i.e., steroids), a medication variable was created to code the likelihood of medication type affecting cortisol levels (0 = unlikely, 1 = somewhat likely, 2 = very likely; *M* = 0.34, *SD* = 0.73; Granger et al., 2009).

#### 2.4.2. Depressive symptoms

Primary participants self-reported depressive symptoms using the 20-item Center for Epidemiological Studies Depression Scale for Children (CES-DC; Weissman et al., 1980). The CES-DC has good psychometric properties and has been validated as a screening tool for individuals from 6 to 23 years of age (Fendrich et al., 1990). Participants reported on a scale of 0 (Not at all) to 3 (A lot) how much they felt or acted a certain way during the past week, and a sum composite was created (α = .90). In the current sample, CES-DC scores ranged from 1 to 45 (*M* = 15.59, *SD* = 10.21). A dichotomous score was created based on the CES-DC cutoff of higher risk for depression (Fendrich et al., 1990; Weissman et al., 1980) to use for analyses given its implications for practice. Participants who scored 15 or above in the current sample were classified as higher risk for depression (*n* = 35 [48.61 %] of children and *n* = 29 [44.62 %] of adolescents). These rates of higher depression risk fall within the range of other studies that have administered the CES-DC in U.S.-based samples of children and adolescents since the COVID-19 pandemic (Gotlib et al., 2021; Miller et al., 2021; Demaray et al., 2022).

#### 2.4.3. Sibling relationship quality

Primary participants completed the Sibling Attachment Inventory (SAI; Noel et al., 2018), a 21-item questionnaire comprised of three subscales: communication (e.g., “I tell my brother or sister about my problems and troubles”; α = .89), trust (e.g., “My brother or sister accepts me as I am”; α = .85), and alienation (e.g., “My brother or sister doesn’t understand my problems”; α = .64). Items were rated on a scale of 1 (Never true) to 3 (Always true) and a mean score was calculated for each of the three subscales (*M* = 2.20, *SD* = 0.48 for communication; *M* = 2.65, *SD* = 0.43 for trust; and *M* = 1.60, *SD* = 0.33 for alienation).

### 2.5. Data analytic plan

Multilevel modeling was conducted in Mplus software (Muthén and Muthén, 2017) to model individuals’ trajectories of cortisol levels. Multilevel modeling is an ideal statistical method for analyzing data that is nested within individuals, accounting for both within- and between-person variability (Hox et al., 2017). Level one of the model represented how individuals’ cortisol levels changed as a function of time relative to stressor onset (start of TSST speech). Time to/from start of TSST speech was calculated for each of the seven cortisol collection timepoints. Linear and quadratic terms were added to the model to represent the rate of change from baseline to peak reactivity and recovery of the cortisol trajectory, respectively. That is, a positive linear term represented the magnitude of the initial increase in cortisol (i.e., the slope of cortisol trajectory at time = 0) and the quadratic coefficient determined the timing and magnitude of cortisol recovery (i.e., the width of the cortisol trajectory arc). This approach allowed us to account for non-uniform timing of cortisol samples across individuals as well as different peak times, as some individuals peaked at 20 min post-TSST start while others peaked at 30 min post-TSST start.

#### 2.5.1. Main analyses

For our main analyses, Level two of the model evaluated between-person differences in cortisol trajectories based on the following predictors: condition (0 = sibling, 1 = stranger), age group (0 = child, 1 = adolescent), and covariates including participant sex (0 = female, 1 = male), time since wake (mean-centered), and medication usage (centered at zero). A condition x age group two-way interaction term was tested in the subsequent model.

#### 2.5.2. Depressive symptoms

We then added depressive symptoms into the model and tested a two-way interaction between condition x depressive symptoms, as well as a three-way interaction between condition x age group x depressive symptoms. Significant interactions were probed for lower- and higher-risk groups (0 = lower risk for depression and 1 = higher risk for depression) using simple slopes analyses.

#### 2.5.3. Sibling relationship quality

Lastly, we conducted multilevel models using only the sample of participants who were in the sibling condition (*n* = 69). SAI domains (communication, trust, alienation) were entered at level two of separate models to evaluate between-person differences in cortisol trajectories based on each domain.

#### 2.5.4. Missing data

Missing data were minimal for cortisol (about 1.36 % across all participants and timepoints) and handled using full information maximum likelihood in Mplus. As for predictors, eleven participants (8.03 % of the full sample) were missing at least some data on depressive symptoms. Because most participants were only missing responses on one or two items, multiple imputation was conducted on an item-level basis using IBM SPSS Statistics Version 28.0. Then, sum composite scores for depressive symptoms were created and averaged across five imputed datasets for each individual with missing data. Multiple imputation was also conducted on an item-level basis for nine participants (6.57 % of the full sample) who were missing data on sibling relationship quality items. Mean composite scores were created for each subscale (communication, trust, and alienation) and averaged across five imputed datasets.

#### 2.5.5. Supplementary analyses

Supplementary materials include additional analyses on baseline cortisol levels, potential sex and gender differences, descriptives for sibling relationship quality, perceived helpfulness as a covariate and predictor, and self-reported stress as an outcome.

### 2.6. Power analysis

Power analysis was conducted using PinT 2.12 (Bosker et al., 1999) – a software for power analysis in two-level models. Based on the standard errors estimated for a two-level model that included random linear and quadratic effects of time at level one, and condition, age group, and the condition x age group interaction at level two, our sample had sufficient power to estimate medium-size (Cohen’s *d* = 0.50) interaction effect for condition x age group interaction predicting the intercept, linear slope and quadratic change (power ranging from .91 to .94), but not for small-size (Cohen’s *d* = 0.20) interaction effects (power ranging from .27 to .33). Power analysis was not conducted for the condition x age group x depressive symptoms three-way interaction model or sibling relationship quality models, as these research questions were exploratory.

## 3. Results

In the unconditional growth model, deviance scores indicated that a quadratic growth curve was a better fit for the cortisol trajectory than a linear growth curve, (Δχ^2^(4) = 1003.42– 876.07 = 127.35, *p* < .001). Both linear and quadratic terms were significant (*b* = 0.53, *SE* = 0.09, *p* < .001 for linear term and *b* = −0.84, *SE* = 0.10, *p* < .001 for quadratic term), confirming an initial rise in cortisol levels, followed by a subsequent decrease over time.

### 3.1. Main analyses

In the conditional growth model, the main effect of condition (sibling versus stranger) was not statistically significant for the linear (*b* = −0.01, *SE* = 0.19, *p* > .10) or quadratic (*b* = 0.10, *SE* = 0.20, *p* > .10) terms. There were also no significant main effects of age group, sex, time since wake, or medication, *p*’s > .05. In the interaction model, the condition x age group two-way interaction term was not significant for the linear (*b* = 0.60, *SE* = 0.35, *p* = .09) or quadratic (*b* = −0.15, *SE* = 0.39, *p* > .10) terms (See Supplemental Table 2).

### 3.2. Depressive symptoms

The condition x depressive symptoms two-way interaction term was not statistically significant for the linear (*b* = −0.07, *SE* = 0.36, *p* > .10) or quadratic (*b* = 0.03, *SE* = 0. 40, *p* > .10) terms.

**Fig. 2.**
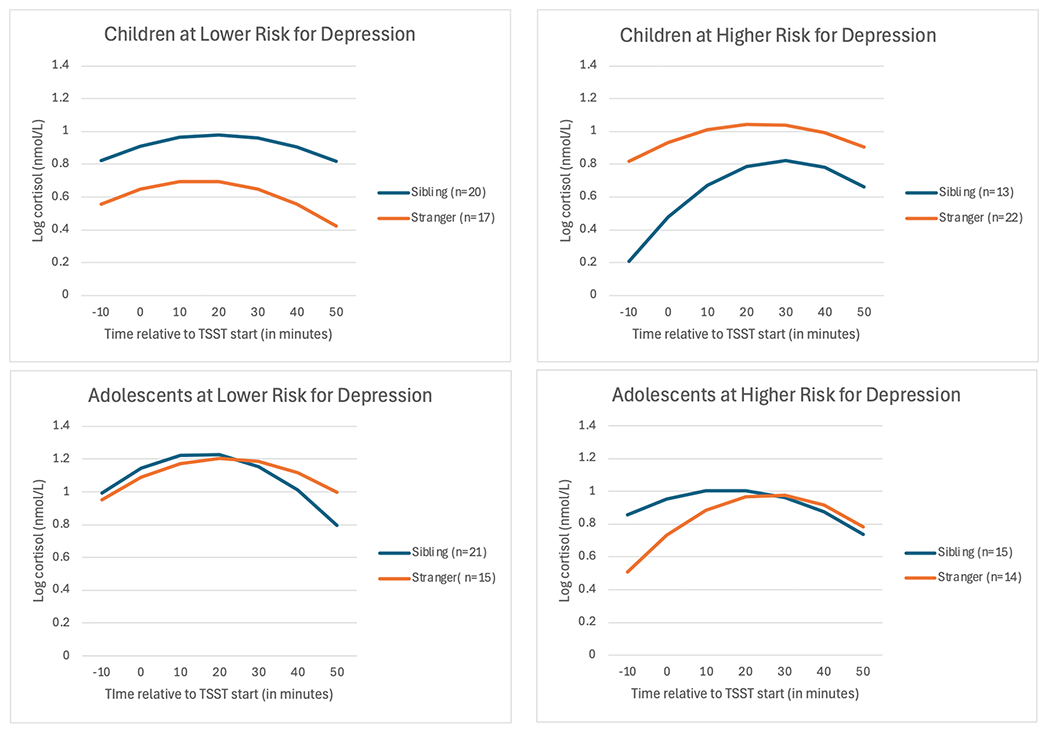
Effect of condition on log cortisol by age group and depressive symptoms. Notes: Cortisol values were measured in nmol/L and natural log-transformed. Individuals who scored ≥ 15 on the CES-DC were classified as higher risk for depression. Adolescents at higher risk for depression showed significantly higher cortisol reactivity in the stranger condition than the sibling condition, *p* < .05. There were no significant differences in cortisol reactivity or recovery by condition for adolescents at lower risk for depression or for children at lower and higher risk for depression.

The condition x age group x depressive symptoms three-way interaction term was statistically significant for the linear (*b* = 1.52, *SE* = 0.73, *p* < .05) and quadratic (*b* = −1.70, *SE* = 0.83, *p* < .05) terms of the cortisol trajectory (see Table 1, Model 1). These interactions were probed further by testing the main effect of condition on cortisol for children and adolescents at higher versus lower risk for depression (see Table 2 for sample sizes by condition, age group, and depression risk). As the baseline levels of cortisol significantly differed for one group (i.e., children at high risk for depression; see Fig. 2 and Supplemental Table 4), follow-up analyses were conducted controlling for the intercept (see Table 1, Model 2). The three-way interaction still remained significant for the linear (*b* = 1.58, *SE* = 0.74, *p* < .05) and quadratic (*b* = −1.92, *SE* = 0.85, *p* < .05) terms after controlling for the intercept, so interactions were probed again for each of the four groups from this new model (See Table 3). Condition significantly predicted the linear term for adolescents at higher risk for depression such that those who prepared with a sibling exhibited significantly lower cortisol reactivity to the stressor than those who prepared with a stranger (*b* = 0.70, *SE* = 0.32, *p* < .05). The effect of condition on the linear term did not reach statistical significance for children at higher risk for depression (*b* = −0.84, *SE* = 0.45, *p* = .06), nor for children and adolescents at lower risk for depression, *p*’s > .10. Additionally, condition did not significantly predict the quadratic term for any of the groups, *p*’s > .10.

### 3.3. Sibling relationship quality

**Fig. 3.**
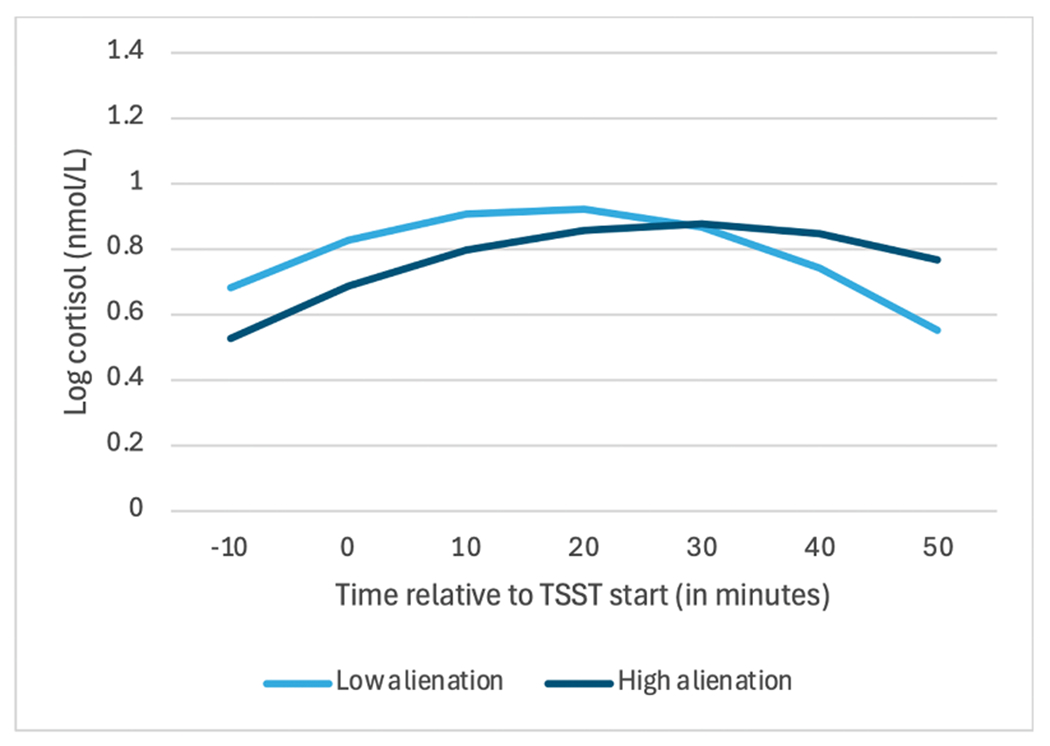
Log cortisol by sibling alienation for sibling condition only (*n*= 69). Notes: Cortisol values were measured in nmol/L and natural log-transformed. Cortisol trajectories were graphed at low (−1 SD below the mean) and high (+1 SD above the mean) levels of alienation. Higher alienation was associated with impaired cortisol recovery post-stressor, *p* < .05.

In the multilevel models for sibling condition only (*n* = 69), communication and trust did not significantly predict the linear or quadratic terms of cortisol trajectories, *p*’s > .10, but alienation significantly predicted the quadratic term (*b* = 0.57, *SE* = 0.28, *p* < .05; See Supplemental Table 3). Cortisol trajectories were graphed at low (−1 SD below the mean) and high (+1 SD above the mean) levels of alienation. Results indicated that higher levels of alienation were associated with impaired cortisol recovery post-stressor (see Fig. 3).

## 4. Discussion

The current study used an experimental paradigm to test the effectiveness of siblings as social buffers during an acute psychosocial stressor compared to strangers. Our findings illustrate a nuanced story of how siblings may (or may not) buffer cortisol responses to stress depending on age and level of depressive symptoms. We also provide some evidence that sibling relationship quality can influence buffering effectiveness. This study advances our understanding of social buffering across development and presents some of the first evidence about how sibling relationships may affect stress physiology in children and adolescents.

Contrary to our primary hypothesis that both children and adolescents would benefit from preparing for the TSST with a sibling, there was no main effect of condition on the cortisol stress response (reactivity or recovery) across the full sample. Furthermore, the condition x age group interaction was not statistically significant, meaning the effect of condition did not differ between children and adolescents. Based on these results, we conclude that siblings did not differ from strangers in affecting cortisol reactivity to and recovery from psychosocial stress for children or adolescents in our sample. However, this finding does not mean that siblings have no buffering potential at all, or that they are unable to buffer against physiological stress for *all* children and adolescents. As extensively discussed in the following paragraphs, factors like depressive symptoms and perceived sibling relationship quality may explain some within-group individual differences.

Importantly, we found that condition and age predicted differing cortisol responses depending on the level of participants’ depressive symptoms. First, siblings did not have any significant effects on the cortisol response compared to strangers for children or adolescents at lower risk for depression. Within the group of adolescents at higher risk for depression (i.e. scored above the CES-DC cutoff), those who prepared for the TSST with a sibling showed lower cortisol reactivity than those who prepared with a stranger. It should be noted that while baseline levels of cortisol seemed to differ by condition for this group, this difference was not statistically significant (see Supplemental Table 4). Thus, one interpretation of this finding is that siblings buffered cortisol reactivity to stress in these individuals. However, given that this group’s sibling-condition trajectory resembled those of sibling and stranger conditions in other groups (see Fig. 2), this finding may alternatively be interpreted as an enhanced stranger reactivity effect rather than a sibling buffering effect. This pattern of results aligns with previous literature (Steger and Kashdan, 2009) on how depressed individuals experience greater sensitivity to negative social events (higher reactivity to the TSST in the stranger condition) but also to positive social interactions (buffering by siblings). In contrast, siblings did not buffer cortisol reactivity for children at higher risk for depression (see Fig. 2). As seen in Table 3, the effect of condition on reactivity for children at higher risk for depression was the largest effect across all groups, suggesting that siblings could actually increase cortisol reactivity compared to strangers. However, this association did not reach statistical significance. Given that the sibling condition of children at higher risk for depression had the smallest sample size of all subgroups (see Table 2), it could be that we did not have enough statistical power to detect an effect.

There are several possible explanations for why siblings may have buffered cortisol reactivity for *adolescents* but not for *children* at higher risk for depression in the current sample. First, the nature of sibling relationships often changes throughout development in ways that align with our findings. For example, McHale et al. (2024) reported that (1) sibling intimacy declines from middle childhood into early adolescence but increases again in late adolescence, and (2) sibling conflict peaks during middle childhood and declines as adolescence progresses. Indeed, in our sample, we found that adolescents reported significantly higher communication and trust and lower alienation with their siblings than children did (see Supplemental material). Furthermore, depressive symptoms interacted with age such that adolescents at higher risk for depression reported the highest levels of communication and trust while children at higher risk for depression reported the lowest levels of communication and trust. The means for communication and trust were significantly different between these groups (see Supplemental Table 8). Based on these findings, it makes sense that adolescents at higher risk for depression may benefit from sibling buffering while children at higher risk for depression do not.

Our findings also partially align with literature on how depression relates to HPA activity in children and adolescents. Studies have reported mixed findings for baseline cortisol levels for children and adolescents with and without high depressive symptoms, with some reporting no differences (Colich et al., 2015; Hankin et al., 2010; Zajkowska et al., 2022) and others reporting both higher (Lopez-Duran et al., 2009) and lower (Badanes et al., 2011; Keenan et al., 2013) baseline cortisol levels for those with high depressive symptoms. In our sample, we did not find that depressive symptoms predicted baseline cortisol levels (see Supplemental Table 5). Depression has also been linked to both hyper- and hypo-reactivity patterns of cortisol in response to stress (Rao et al., 2008; Stewart et al., 2013; Luby et al., 2003, 2004). Different types of depressive symptom profiles (e.g., affective vs. neurovegetative) and depressive symptom severity may predict heterogeneity in cortisol levels and reactivity in youth (Morris et al., 2017; Harkness et al., 2011). Developmental timing is another factor that may explain this heterogeneity of findings, with pre-pubertal children at high risk of depression exhibiting blunted responses and post-pubertal adolescents at high risk of depression exhibiting increased reactivity (Hankin et al., 2010; Colich et al., 2015). These previous findings could explain why in our sample, adolescents at higher risk for depression showed substantially increased reactivity to the TSST when they prepared with a stranger compared with a sibling. However, this explanation does not hold for children at higher risk for depression, who did not exhibit blunted cortisol reactivity in either condition.

We did not find any significant main or interactive effects of condition for cortisol recovery. While it is possible that siblings truly do not buffer recovery, this null effect may also be due to study design. Unlike previous studies where participants reunited with their social partners post-TSST (Doom et al., 2015, 2017), siblings in our study were not present post-TSST due to the online format and to reduce burden on the siblings who were participating. Primary participants only interacted with the experimenter (not parents or siblings) during the recovery portion. Furthermore, studies that have reported effective social buffering of cortisol recovery were designed such that the social partner interacted with participants post-TSST rather than before the task (Seltzer et al., 2010; Calhoun et al., 2014). Thus, it seems that timing of support may matter for different components of the cortisol stress response, and research on sibling buffering should be replicated with different study designs (e.g., where the sibling provides support post-stressor).

We also tested whether self-reported sibling relationship quality influenced the cortisol stress response for those who prepared with a sibling (*n* = 69). While communication and trust did not significantly predict cortisol reactivity or recovery, we found that higher sibling alienation predicted impaired cortisol recovery (i.e., slower return to baseline). This finding is consistent with previous work where negative friendship qualities, but not positive qualities, predicted impaired cortisol recovery in adolescents (Calhoun et al., 2014). Although we found some evidence that self-reported sibling relationship quality was associated with cortisol recovery, it remains to be explored whether differences in *observed* quality of sibling interactions during the study session can affect cortisol reactivity or recovery (see Future Directions).

### 4.1. Study limitations and future directions

There were limitations to the current study. First, due to low statistical power, the results of the condition x age group x depressive symptoms three-way interaction should be interpreted with caution and must be replicated. Second, as this study was conducted virtually in participants’ homes, there were some interruptions to the study protocol that may have lowered the effectiveness of the TSST-OL (e.g., family members entering participants’ room, noises outside of room, technological difficulties). However, analyses suggested that the TSST-OL reliably increased cortisol levels overall. Third, the majority of our sample identified as non-Hispanic White and came from middle-to-high socioeconomic status households, meaning our findings likely cannot be generalized to more racially, ethnically, or socioeconomically diverse groups. Broadening the diversity of samples in this research is especially important as the nature of sibling relationships varies across cultures (McHale et al., 2012; Updegraff et al., 2011). Cross-cultural differences in sibling relationships (e.g., participants’ beliefs about sibling roles and expectations across cultures) should be explored in future studies. Finally, while this study tested two different age groups using an experimental design, the study was still cross-sectional rather than longitudinal. Thus, we cannot make definitive conclusions about the developmental trajectory of social buffering by siblings over time in the same individuals. Future longitudinal studies should be conducted that test for within-person differences in social buffering across development.

It is possible that the statistically null condition and condition x age effects in the full sample are due to the use of the TSST-OL in the current study. Recent research suggests that while virtual TSSTs are effective at eliciting physiological stress responses, which we also observed in this study, they may not be as potent for increasing cortisol levels as in-person TSSTs. It is possible that we could have observed a stronger main effect of condition or a clearer condition x age group interaction effect if participants completed the in-person TSST in a standard lab environment. There is also evidence in children that in-person, full-contact social support is more potent than verbal support only for reducing cortisol reactivity (Seltzer et al., 2010). As a result, the effect of the sibling condition on cortisol reactivity could have been larger if speech preparation were conducted in person. Although this study was conducted virtually given the context of the pandemic that made in-person research more difficult, it is an important first step in understanding the potential effect of sibling buffering. We encourage others to test the impact of in-person sibling social buffering in future studies.

The current study’s design only tested whether the *presence* of siblings during the speech prep portion was more beneficial than that of a stranger, as we did not formally test anything about what siblings did during speech prep. For example, it is unclear whether siblings buffered stress by offering practical support (e.g., help crafting the speech) or whether their mere presence and familiarity (e.g., having discussions unrelated to the task) was enough for participants to feel less stressed. It is also possible that some siblings were *not* supportive and may have induced more stress for participants. To get closer at these mechanisms of support (or conflict), an important next step is to examine whether sibling buffering effectiveness differs by indices of *observed* sibling-participant interaction quality during the speech prep phase, such as what type of support siblings offered (e.g., advice, encouragement), how much participants agreed with their siblings, and any negative interactions that occurred (e.g., criticism or disagreement between siblings).

Social buffering depends on the context of both the stressor and the support given. For example, recent research suggests that youth who undergo the TSST alone but prepare with a friend show heightened cortisol reactivity to the TSST. However, if the friend also experiences the stressor (“shares the load”), the youth’s cortisol stress responses is then buffered (Filetti et al., 2025). Due to shared home, school, and community environments, siblings can often experience the same or similar stressors, sometimes even simultaneously (e.g., parental conflict, transition to a new home or school). Thus, it is possible that social buffering between siblings may be more effective in contexts where they “share the load” of a stressor together, as compared to when a sibling provides support but does not undergo the stressor themselves. Furthermore, while the current study used the TSST to experimentally induce social evaluative threat, perhaps results would differ in the context of more ecologically valid stressors. For example, Adams et al. (2011) found that having a best friend present during a negative or stressful event outside of the lab buffered the effect of that experience on cortisol reactivity. These possibilities should be tested in future research.

Despite the limitations noted above, the current study lays the groundwork for future research on social buffering in siblings, for which we provide specific recommendations below. Our study limited participants to siblings of the same age or older, though researchers may want to include younger siblings in future studies, as some research suggests that individuals equally benefit from older and younger sibling support (Branje et al., 2004). It is also important to consider intersections between gender and age in sibling pairs, as previous research suggests that sibling relationship qualities vary based on gender and age constellations (Buist et al., 2013; Milevsky, 2021). For example, older brother/younger sister pairs often report the lowest intimacy out of all possible constellations (Aguilar et al., 2001; Buist, 2010). Finally, while the current study mostly included biological siblings, future researchers may consider testing similar effects in other sibling types (e.g., step-siblings, adopted siblings), which will require more targeted sampling of these families.

To our knowledge, this is one of the first studies to document differences in social buffering effectiveness by level of participants’ depressive symptoms. Future studies are needed to replicate this effect and also to clarify the mechanisms by which depression affects social buffering of the HPA axis. For example, it could be that depressed individuals may seek out social support more than non-depressed individuals, which may also differ by age. It would be beneficial to look at observations of how much participants solicited or accepted support across conditions by level of depressive symptoms. Furthermore, while the current study was cross-sectional and only measured concurrent depression, longitudinal studies can follow individuals over time and examine how social buffering of HPA axis may be related to later depression (e.g., high levels of buffering could protect against future symptoms).

### 4.2. Conclusion

The current study found that for most groups of children and adolescents in our sample, siblings were not more effective buffers against cortisol increases in response to psychosocial stress than strangers. However, we provide preliminary evidence that siblings may buffer the cortisol stress response compared to strangers for adolescents at higher risk of depression. This finding is particularly encouraging given previous research demonstrating reduced effectiveness of parents and friends as social buffers of the cortisol stress response during adolescence (Hostinar et al., 2015a; Doom et al., 2015, 2017). Our results suggest that while not everyone may benefit from siblings reducing cortisol responses to stress, it may be worthwhile to leverage siblings in prevention and intervention efforts for adolescents who are at high risk of depression. We also provide preliminary evidence that lower feelings of alienation in the sibling relationship could improve cortisol recovery post-stressor in the context of preparing for a stressor with a sibling. This preliminary finding suggests that enhancing sibling relationship quality, even for individuals who are not at high risk for depression, could be beneficial for supporting recovery from acute stressors. Future research is needed to replicate our findings, as well as advance our understanding of sibling social buffering. In particular, further research is needed to clarify the mechanisms by which siblings can buffer the HPA axis against psychosocial stress and how depressive symptoms affect social buffering processes.

## Supplementary Material

1

## Figures and Tables

**Table 1:** Condition x age group x depressive symptoms three-way interaction model for log cortisol.

	Model 1: Unadjusted log cortisol trajectories *b* (SE)	Model 2: Log cortisol trajectories, controlling for cortisol intercept *b* (SE)
**Fixed effects**		
**Intercept** ^a^	0.91 (0.12)***	0.91 (0.12)***
Condition	−0.26 (0.20)	−0.26 (0.20)
Age group	0.23 (0.16)	0.23 (0.16)
Sex	0.36 (0.11)**	0.36 (0.11)**
Time since wake	−0.03 (0.02)	−0.03 (0.02)
Medication	−0.05 (0.06)	−0.05 (0.06)
Depressive symptoms	−0.43 (0.20)*	−0.43 (0.20)*
Condition x age group	0.21 (0.30)	0.21 (0.30)
Condition x depressive	0.72 (0.31)*	0.72 (0.31)*
Age group x depressive	0.24 (0.25)	0.24 (0.25)
Condition x age x depressive	−0.88 (0.42)*	−0.88 (0.42)*
**Linear slope**	0.42 (0.30)	0.35 (0.32)
Condition	−0.01 (0.35)	0.01 (0.35)
Age group	0.26 (0.34)	0.24 (0.35)
Sex	−0.15 (0.19)	−0.17 (0.19)
Time since wake	0.04 (0.04)	0.04 (0.04)
Medication	−0.20 (0.15)	−0.20 (0.15)
Depressive symptoms	0.96 (0.48)*	0.99 (0.48)*
Condition x age group	−0.02 (0.46)	−0.04 (0.46)
Condition x depressive	−0.80 (0.58)	−0.85 (0.58)
Age group x depressive	−1.21 (0.55)*	−1.22 (0.55)*
Condition x age x depressive	1.52 (0.73)*	1.58 (0.74)*
Intercept of log cortisol	–	0.07 (0.14)
**Quadratic growth**	−0.64 (0.30)*	−0.41 (0.37)
Condition	−0.18 (0.39)	−0.24 (0.39)
Age group	−0.68 (0.34)*	−0.62 (0.34)^†^
Sex	0.13 (0.21)	0.22 (0.20)
Time since wake	−0.09 (0.05)^†^	−0.10 (0.05)*
Medication	0.19 (0.15)	0.18 (0.15)
Depressive symptoms	−0.76 (0.54)	−0.87 (0.55)
Condition x age group	0.58 (0.49)	0.63 (0.48)
Condition x depressive	0.85 (0.65)	1.03 (0.67)
Age group x depressive	1.25 (0.63)*	1.31 (0.63)*
Condition x age x depressive	−1.70 (0.83)*	−1.92 (0.85)*
Intercept of log cortisol	–	−0.25 (0.15)^†^
**Random effects**		
**Residual variance**	0.06 (0.01)***	0.06 (0.01)***
Intercept	0.33 (0.05)***	0.33 (0.05)***
Linear slope	0.67 (0.11)***	0.67 (0.11)***
Quadratic growth	0.46 (0.16)**	0.44 (0.16)**

Notes: Cortisol values were measured in nmol/L and natural log-transformed. Condition was coded as 0 = sibling and 1 = stranger. Age group was coded as 0 = child and 1 = adolescent. Sex was coded as 0 = female and 1 = male. Depressive symptoms was coded as 0 = lower risk of depression and 1 = higher risk of depression. Time since wake was mean-centered and medication was centered at zero.

a Intercept represents log cortisol values for sibling condition at TSST start (T + 0 min).

* *p* < .05

** *p* < .01

*** *p* < .001

† *p* < .10

**Table 2:** Sample size by condition, age group, and depression risk.

		Condition		TOTAL
		Sibling	Stranger	
**Children (9–11 years)**	Lower risk for depression	20	17	37
	Higher risk for depression	13	22	35
**Adolescents (15–17 years)**	Lower risk for depression	21	15	36
	Higher risk for depression	15	14	29
**TOTAL**		69	68	137

Notes: Individuals who scored ≥ 15 on the CES-DC were classified as higher risk for depression based on previously published cut-offs (Fendrich et al., 1990; Weissman et al., 1980).

**Table 3:** Simple slope analyses: main effect of condition on log cortisol by age group and depressive symptoms.

	Children (9–11 years)		Adolescents (15–17 years)	
	Lower risk for depression *b* (SE)	Higher risk for depression *b* (SE)	Lower risk for depression *b* (SE)	Higher risk for depression *b* (SE)
**Intercept** ^a^				
Condition	−0.26 (0.20)	0.45 (0.24)^†^	−0.06 (0.21)	−0.22 (0.17)
**Linear slope**				
Condition	0.01 (0.35)	−0.84 (0.45)^†^	−0.03 (0.28)	0.70 (0.32)*
**Quadratic growth**				
Condition	−0.24 (0.39)	0.79 (0.52)	0.39 (0.27)	−0.50 (0.39)

Notes: Cortisol values were measured in nmol/L and natural log-transformed. Condition was coded as 0 = sibling and 1 = stranger. Individuals who scored ≥ 15 on the CES-DC were classified as higher risk for depression.

a Intercept represents log cortisol values for sibling condition at TSST start (T + 0 min).

* *p* < .05

** *p* < .01

*** *p* < .001

† *p* < .10

## Footnotes

1 Though the World Health Organization characterizes adolescence starting at the age of 10, we acknowledge that our “children” group may include individuals from late childhood and early adolescence (pre-puberty). We call this group “children” for the sake of simplicity.

## Appendix A. Supporting information

Supplementary data associated with this article can be found in the online version at doi:10.1016/j.psyneuen.2025.107580.
