# Supplementary material for: Social buffering by siblings in childhood and adolescence
Source: Psychoneuroendocrinology. Author manuscript; Available in PMC 2026 Jul 27. (PMC13403998; doi:10.1016/j.psyneuen.2025.107580)
Supplement: 1 [file NIHMS2181336-supplement-1.docx]

**Supplemental Methods**

**Measures**

**Self-reported stress.** At the end of the session, primary participants used a 5-point Likert scale (1= calm, 2= low stress, 3= medium stress, 4= somewhat stressed, 5= very high stress) to rate how stressed they felt at five different timepoints: arrival, speech preparation, speech delivery, math task, and end of session.

**Perceived helpfulness.** After reporting on their stress levels, primary participants were asked to rate on a scale of 1 (Not at all) to 5 (Extremely) how helpful the experimenter or sibling was when they were preparing their speech.

**Data analytic plan**

**Baseline levels of cortisol.** Independent samples t-tests were conducted to examine whether baseline levels of cortisol (at the first collection timepoint) significantly differed by condition within each of the four age group x depressive symptoms groups (i.e., children and adolescents at higher and lower risk of depression).

**Main effects of depressive symptoms.** Multilevel modeling was conducted to test depressive symptoms as a predictor of baseline cortisol, reactivity, and recovery, controlling for condition, age, and other covariates (participant sex, medication usage, and time since wake).

**Participant sex and sibling gender match as moderators of condition on cortisol.** As there is mixed evidence for sex or gender differences in social buffering effectiveness (Gunnar, 2017), we tested whether the effects of condition and condition x age differed by primary participant sex (0= female, 1= male), as well as by sibling “gender match” (i.e., whether participant gender matched their sibling gender; 0= different gender, 1= same gender). For these aims, we tested two-way interactions between condition x sex and condition x gender match at level two of the multilevel model for cortisol trajectories. We also tested three-way interactions between condition x age group x sex interaction and condition x age group x gender match. Of note, we did not test 4-way interactions between condition x age group x depressive symptoms x sex/gender match due to power concerns and because we did not expect sex or gender match to interact with depressive symptoms.

**Sibling relationship quality descriptives.** We conducted three separate three-way ANOVAs to examine whether SAI domains (communication, trust, alienation) differed by condition x age group x depressive symptoms. Post-hoc pairwise comparisons using the Bonferroni correction were conducted to test between-group differences.

**Perceived helpfulness descriptives.** As with SAI domains (see above), we conducted a three-way ANOVA to examine whether perceived helpfulness ratings differed by condition x age group x depressive symptoms. Post-hoc pairwise comparisons using the Bonferroni correction were conducted to test between-group differences.

**Perceived helpfulness as a covariate in main model for cortisol.** Perceived helpfulness was included as a covariate in our three-way condition x age group x depressive symptoms multilevel model for cortisol trajectories, to test whether our main finding became non-significant after controlling for variance in perceived helpfulness of sibling and stranger.

**Perceived helpfulness as a predictor of sibling buffering.** We also tested perceived helpfulness as a predictor of cortisol reactivity and recovery for those in the sibling condition only (*n*= 69).

**Self-reported stress as an outcome.** Lastly, we examined whether social buffering effects were observed for self-reported stress. Missing data were minimal for self-reported stress (about 1.02%) and handled using full information maximum likelihood in Mplus. Analyses for self-reported stress mirrored those of cortisol trajectories. Level one of the multilevel models represented how individuals’ perceived stress levels changed across the five timepoints. Level two of the model evaluated between-person differences in self-reported stress trajectories based on the following predictors: condition (0= sibling, 1= stranger), age group (0= child, 1= adolescent), and primary participant sex as a covariate (0= female, 1= male). The interaction model included a condition x age group interaction term. We then added depressive symptoms into the model and tested a two-way interaction between condition x depressive symptoms, as well as a three-way interaction between condition x age group x depressive symptoms.

**Supplemental Results**

**Baseline levels of cortisol**

Baseline cortisol levels did not significantly differ by condition for children at lower risk for depression, *t*(35)= 1.60, *p* >.10, adolescents at lower risk for depression, *t*(34)= .44, *p* >.10, or adolescents at higher risk for depression, *t*(27)= 1.38, *p* >.10. For children at higher risk for depression, those who prepared for the TSST with a sibling had significantly lower levels of baseline cortisol than those who prepared with a stranger, *t*(33)= -2.18, *p* <.05. See Supplemental Table 4 for means by condition, age group, and depressive symptoms.

**Main effects of depressive symptoms**

Depressive symptoms did not significantly predict the linear or quadratic terms of cortisol trajectories, *p*’s >.10 (See Supplemental Table 5).

**Participant sex and sibling gender match as moderators of condition on cortisol**

All two-way (condition x sex/gender match) and three-way interaction terms (condition x age group x sex/gender match) that we tested were not statistically significant for linear or quadratic terms of the cortisol trajectories, *p’*s >.10. See Supplemental 6 for results from multilevel models with primary participant sex and Supplemental Table 7 for results from multilevel models with sibling gender match.

**Sibling relationship quality descriptives.**

**Communication.** The three-way interaction between condition x age group x depressive symptoms was not significant for communication, *F*(1, 129)= .03, *p* >.10. However, there was a significant two-way interaction between age group x depressive symptoms, *F*(1, 129)= 5.76, *p* <.05. Adolescents reported significantly higher levels of communication with their sibling than children, *F*(1, 129)= 20.87, *p* <.001. Individuals at lower risk for depression reported significantly higher levels of communication than individuals at higher risk for depression, *F*(1, 129)= 4.16, *p* <.05. Within the child age group, those at lower risk for depression reported significantly higher levels of communication than those at higher risk for depression, *F*(1, 129)= 10.31, *p* <.01, but adolescents at higher and lower risk for depression did not significantly differ from each other, *F*(1, 129)= .06, *p* >.10. Within the higher risk for depression group, adolescents reported significantly higher levels of communication than children, *F*(1, 129)= 22.54, *p* <.001, but children and adolescents at lower risk for depression did not significantly differ from each other, *F*(1, 129)= 2.55, *p* >.10. See Supplemental Table 8 for means by age group and depressive symptoms.

**Trust.** The three-way interaction between condition x age group x depressive symptoms was not significant for trust, *F*(1, 129)= .03, *p* >.10. However, there was a significant two-way interaction between age group x depressive symptoms, *F*(1, 129)= 10.27, *p* <.01. Adolescents reported significantly higher levels of trust with their sibling than children, *F*(1, 129)= 22.67, *p* <.001. Individuals at lower risk for depression reported significantly higher levels of trust than individuals at higher risk for depression, *F*(1, 129)= 7.03, *p* <.01. Within the child age group, those at lower risk for depression reported significantly higher levels of trust than those at higher risk for depression, *F*(1, 129)= 17.93, *p* <.001, but adolescents at higher and lower risk for depression did not significantly differ from each other, *F*(1, 129)= .15, *p* >.10. Within the higher risk for depression group, adolescents reported significantly higher levels of trust than children, *F*(1, 129)= 29.45, *p* <.001, but children and adolescents at lower risk for depression did not significantly differ from each other, *F*(1, 129)= 1.31, *p* >.10. See Supplemental Table 8 for means by age group and depressive symptoms.

**Alienation.** The three-way interaction between condition x age group x depressive symptoms was not significant for alienation, *F*(1, 129)= 1.15, *p* >.10. However, there were significant effects of age group and depressive symptoms. Adolescents reported significantly lower levels of alienation with their sibling than children, *F*(1, 129)= 5.99, *p* <.05. Individuals at lower risk for depression reported significantly lower levels of alienation than individuals at higher risk for depression, *F*(1, 129)= 13.72, *p* <.001. See Supplemental Table 8 for means by age group and depressive symptoms.

**Perceived helpfulness descriptives**

The three-way interaction between condition x age group x depressive symptoms was not statistically significant for perceived helpfulness, *F*(1, 129)= 1.63, *p* >.10. However, there was a significant effect of condition. Participants rated siblings (*M*= 3.54, *SE*= .14) as more helpful than strangers (*M*= 2.81, *SE*= .14), *p* <.001.

**Perceived helpfulness as a covariate in main model for cortisol**

After adding perceived helpfulness as a covariate, the three-way interaction between condition x age group x depressive symptoms remained statistically significant for the linear (*b*= 1.60, *SE*= .74, *p <*.05) and quadratic (*b*= -1.89, *SE*= .84, *p* <.05) terms of cortisol trajectories (See Supplemental Table 9).

**Perceived helpfulness as predictor of sibling buffering**

In the multilevel model for sibling condition only (*n*= 69), perceived helpfulness did not significantly predict the linear or quadratic terms of cortisol trajectories, *p*’s >.10 (see Supplemental Table 3).

**Self-reported stress as an outcome**

Overall, participants displayed increases in self-reported stress levels during the test portion and then returned back to baseline: arrival (*M*= 2.41, *SD*= 1.11), speech preparation (*M*= 2.85, *SD*= 1.24), speech delivery (*M*= 4.17, *SD*= 0.94), math task (*M*= 4.02, *SD*= 1.06), and end of session (*M*= 1.75, *SD*= 0.92). Self-reported stress was better modeled via a quadratic growth curve than a linear growth curve (Δχ^2^(4)= 6751.32–2024.05 = 4727.27, *p* <.001). Both linear and quadratic terms were statistically significant in the unconditional growth model (*b*= 1.95, *SE*= .08, *p* <.001 for linear term and *b*= -0.49, *SE*= .02, *p* <.001 for quadratic term), confirming an initial rise and subsequent decrease of self-reported stress across the session.

The conditional growth model showed no statistically significant effects of condition on linear (*b*= 0.22, *SE*= .16, *p* >.10) or quadratic (*b*= -0.05, *SE*= .04, *p* >.10) terms. There were also no statistically significant main effects of age group or sex, *p’*s > .05. All two-way (condition x age, condition x depressive symptoms) and three-way (condition x age x depressive symptoms) interaction terms that we tested were not statistically significant for linear or quadratic terms, *p’*s >.10 (see Supplemental Table 10).

**Summary of Supplemental Results**

- Baseline levels of cortisol significantly differed by condition for children at higher risk for depression (such that those in the sibling condition had significantly lower levels of baseline cortisol than those in the stranger condition) but not for any other groups.
- Primary participant sex and sibling gender match did not significantly moderate the condition or condition x age effects for cortisol reactivity or recovery.
- Sibling relationship quality domains (communication, trust, alienation) significantly differed by age group and depressive symptoms but not by condition. Adolescents at higher risk for depression reported higher communication and trust than children at higher risk for depression.
- Perceived helpfulness significantly differed by condition (such that siblings were rated more helpful than strangers) but not by any other variables.
- Adding perceived helpfulness as a covariate did not nullify the three-way interaction between condition x age group x depressive symptoms for cortisol reactivity.
- Perceived helpfulness did not significantly predict cortisol reactivity or recovery for those in the sibling condition (i.e., did not influence sibling buffering effectiveness).
- There were no significant effects of condition, age group, depressive symptoms or their interactions for self-reported stress trajectories over time.

**Supplemental Tables**

**Supplemental Table 1. Demographic and descriptive data (N= 137)**

| Variable | N (%) or Mean (SD) |
| --- | --- |
| Participant age in years, M (SD) |  |
| Children | 9.81 (0.64) |
| Adolescents | 15.75 (0.75) |
| Participant sex, N (%) |  |
| Female | 67 (48.91%) |
| Male | 70 (51.09%) |
| Participant self-reported gender, N (%)^a^ |  |
| Girl | 64 (46.72%) |
| Boy | 67 (48.91%) |
| Non-binary | 3 (2.19%) |
| Transgender boy | 2 (1.46%) |
| Not reported | 1 (0.73%) |
| Participant race/ethnicity, N (%) |  |
| Non-Hispanic/Latinx White | 102 (74.45%) |
| Hispanic/Latinx | 19 (13.87%) |
| Asian | 12 (8.76%) |
| Other | 4 (2.92%) |
| Sibling age in years, M (SD) | 15. 06 (3.36) |
| Sibling age difference in years, M (SD) | 2.43 (0.95) |
| Sibling self-reported gender, N (%)^b^ |  |
| Boy/Man | 61 (44.53%) |
| Girl/Woman | 66 (48.18%) |
| Non-binary | 7 (5.11%) |
| Transgender girl/woman | 1 (0.73%) |
| Transgender boy/man | 2 (1.46%) |
| Sibling type, N (%) |  |
| Full biological | 125 (91.24%) |
| Half-siblings | 5 (3.65%) |
| Adoptive | 3 (2.19%) |
| Not reported^c^ | 4 (2.92%) |
| Participant-sibling gender match, N (%) | 67 (48.91%) |
| Parent education level, N (%) |  |
| Completed high school or GED | 7 (5.11%) |
| Associate’s degree or college  academia program | 11 (8.03%) |
| Bachelor’s degree | 59 (43.07%) |
| Graduate degree (Master’s or  beyond) | 59 (43.07%) |
| Not reported | 1 (0.73%) |
| Family income level, N (%) |  |
| $50,000 or less | 5 (3.65%) |
| $50,001 - $100,000 | 11 (8.03%) |
| $100,001 - $150,000 | 35 (25.55%) |
| $150,001 - $200,00 | 37 (27.01%) |
| $200,001 or more | 44 (32.12%) |
| Not reported | 5 (3.65%) |

Notes: ^a^ Since primary participants were under 18, we report gender as boy/girl/non-binary. ^b^ As we did not collect reports of sibling biological sex, we only report on their self-reported gender. Since siblings could be either under 18 or 18 and older, we report gender as boy/man, girl/woman, and non-binary. ^c^ The question on sibling type was added into the survey after the first few participants, so a few participants do not have data for this question.

**Supplemental Table 2. Condition x age group two-way interaction model for log cortisol**

|  | *b* (SE) |
| --- | --- |
| Fixed effects |  |
| Intercept^a^ | 0.76 (0.12)*** |
| Condition | 0.06 (0.15) |
| Age group | 0.33 (0.13)* |
| Condition x Age group | -0.22 (0.21) |
| Sex | 0.35 (0.11)** |
| Time since wake | -0.03 (0.02) |
| Medication | -0.09 (0.07) |
| Linear slope | 0.77 (0.29)** |
| Condition | -0.29 (0.29) |
| Age group | -0.22 (0.28) |
| Condition x Age group | 0.60 (0.35)^†^ |
| Sex | -0.12 (0.19) |
| Time since wake | 0.05 (0.04) |
| Medication | -0.15 (0.14) |
| Quadratic growth | -0.90 (0.30)** |
| Condition | 0.17 (0.31) |
| Age group | -0.18 (0.29) |
| Condition x Age group | -0.15 (0.39) |
| Sex | 0.09 (0.21) |
| Time since wake | -0.10 (0.05)^†^ |
| Medication | 0.16 (0.14) |
| Random effects |  |
| Residual variance | 0.06 (0.01)*** |
| Intercept | 0.35 (0.05)*** |
| Linear slope | 0.73 (0.13)*** |
| Quadratic growth | 0.52 (0.19)** |

Notes: Cortisol values were measured in nmol/L and natural log-transformed. Condition was coded as 0 = sibling and 1 = stranger. Age group was coded as 0 = child and 1 = adolescent. Sex was coded as 0 = female and 1 = male. Time since wake was mean-centered and medication was centered at zero. ^a^ Intercept represents log cortisol values for sibling condition at TSST start (T+0 minutes). **p* <.05 ***p* <.01 ****p* <.001 ^†^*p* <.10

**Supplemental Table 3. Sibling Attachment Inventory (SAI) domains and perceived helpfulness as predictors of log cortisol in sibling condition only (*n*= 69)**

|  | SAI subscale: Communication | SAI subscale: Trust | SAI subscale: Alienation | Perceived helpfulness |
| --- | --- | --- | --- | --- |
|  | ***b* (SE)** | ***b* (SE)** | ***b* (SE)** | ***b* (SE)** |
| Fixed effects |  |  |  |  |
| Intercept^a^ | 0.73 (0.13)*** | 0.74 (0.13)*** | 0.76 (0.13)*** | 0.75 (0.13)*** |
| Predictor | -0.03 (0.16) | 0.07 (0.17) | -0.22 (0.15) | 0.07 (0.06) |
| Age group | 0.38 (0.13)** | 0.36 (0.13)** | 0.35 (0.13)** | 0.37 (0.12)** |
| Sex | 0.35 (0.13)** | 0.35 (0.13)** | 0.32 (0.13)* | 0.31 (0.12)** |
| Time since wake | 0.02 (0.03) | 0.02 (0.03) | 0.02 (0.03) | 0.02 (0.03) |
| Medication | -0.06 (0.07) | -0.06 (0.07) | -0.06 (0.07) | -0.06 (0.07) |
| Linear slope | 0.80 (0.32)* | 0.77 (0.32)* | 0.81 (0.34)* | 0.80 (0.34)* |
| Predictor | -0.07 (0.32) | -0.43 (0.35) | -0.04 (0.31) | -0.05 (0.13) |
| Age group | -0.16 (0.28) | -0.90 (0.28) | -0.18 (0.28) | -0.18 (0.28) |
| Sex | -0.19 (0.29) | -0.22 (0.30) | -0.19 (0.29) | -0.17 (0.29) |
| Time since wake | 0.09 (0.06) | 0.08 (0.06) | 0.09 (0.06) | 0.09 (0.06) |
| Medication | -0.23 (0.19) | -0.21 (0.20) | -0.23 (0.19) | -0.23 (0.19) |
| Quadratic growth | -0.96 (0.32)** | -0.93 (0.31)** | -1.03 (0.35)** | -0.96 (0.34)** |
| Predictor | 0.05 (0.36) | 0.48 (0.40) | 0.57 (0.28)* | 0.04 (0.14) |
| Age group | -0.25 (0.27) | -0.34 (0.28) | -0.20 (0.28) | -0.24 (0.28) |
| Sex | 0.19 (0.32) | 0.23 (0.33) | 0.26 (0.32) | 0.17 (0.32) |
| Time since wake | -0.15 (0.07)* | -0.14 (0.07)* | -0.14 (0.07)* | -0.15 (0.07)* |
| Medication | 0.30 (0.19) | 0.29 (0.20) | 0.32 (0.18)^†^ | 0.30 (0.19) |
| Random effects |  |  |  |  |
| Residual variance | 0.06 (0.01)*** | 0.06 (0.01)*** | 0.06 (0.01)*** | 0.06 (0.01)*** |
| Intercept | 0.23 (0.05)*** | 0.26 (0.05)*** | 0.25 (0.05)*** | 0.25 (0.05)*** |
| Linear slope | 0.95 (0.22)*** | 0.92 (0.20)*** | 0.95 (0.22)*** | 0.95 (0.22)*** |
| Quadratic growth | 0.81 (0.30)** | 0.77 (0.26)** | 0.76 (0.30)* | 0.81 (0.30)** |

Notes: Cortisol values were measured in nmol/L and natural log-transformed. Age group was coded as 0 = child and 1 = adolescent. Sex was coded as 0 = female and 1 = male. Time since wake was mean-centered and medication was centered at zero. ^a^ Intercept represents log cortisol values for sibling condition at TSST start (T+0 minutes). **p* <.05 ***p* <.01 ****p* <.001 ^†^*p* <.10

**Supplemental Table 4. Baseline log cortisol levels by condition, age group, and depressive symptoms**

|  | Children (9-11 years) | | Adolescents (15-17 years) | |
| --- | --- | --- | --- | --- |
|  | **Lower risk for depression** | **Higher risk for depression** | **Lower risk for depression** | **Higher risk for depression** |
|  | **Mean (SD)** | **Mean (SD)** | **Mean (SD)** | **Mean (SD)** |
| Sibling | 1.05 (0.59) | 0.31 (0.86)^a^ | 1.24 (0.63) | 1.04 (0.32) |
| Stranger | 0.74 (0.59) | 0.94 (0.82)^a^ | 1.15 (0.66) | 0.75 (0.74) |

Notes: Cortisol values were measured in nmol/L and natural log-transformed. Individuals who scored ≥15 on the CES-DC were classified as higher risk for depression. ^a^ Within children at higher risk for depression, those who prepared for the TSST with a sibling had significantly lower levels of baseline cortisol than those who prepared with a stranger.

**Supplemental Table 5. Main effects of depressive symptoms for log cortisol**

|  | *b* (SE) |
| --- | --- |
| Fixed effects |  |
| Intercept^a^ | 0.86 (0.11)*** |
| Condition | -0.02 (0.11) |
| Age group | 0.22 (0.10)* |
| Sex | 0.36 (0.10)** |
| Time since wake | -0.03 (0.03) |
| Medication | -0.07 (0.07) |
| Depressive symptoms | -0.14 (0.11) |
| Linear slope | 0.52 (0.25)* |
| Condition | -0.05 (0.19) |
| Age group | 0.08 (0.19) |
| Sex | -0.15 (0.19) |
| Time since wake | 0.04 (0.04) |
| Medication | -0.20 (0.15) |
| Depressive symptoms | 0.29 (0.19) |
| Quadratic growth | -0.84 (0.26)** |
| Condition | 0.11 (0.21) |
| Age group | -0.25 (0.19) |
| Sex | 0.10 (0.21) |
| Time since wake | -0.10 (0.05)^†^ |
| Medication | 0.18 (0.14) |
| Depressive symptoms | -0.10 (0.21) |
| Random effects |  |
| Residual variance | 0.06 (0.01)*** |
| Intercept | 0.35 (0.05)*** |
| Linear slope | 0.73 (0.13)*** |
| Quadratic growth | 0.52 (0.18)** |

Notes: Cortisol values were measured in nmol/L and natural log-transformed. Condition was coded as 0 = sibling and 1 = stranger. Age group was coded as 0 = child and 1 = adolescent. Sex was coded as 0 = female and 1 = male. Depressive symptoms was coded as 0 = lower risk of depression and 1 = higher risk of depression. Time since wake was mean-centered and medication was centered at zero. ^a^ Intercept represents log cortisol values for sibling condition at TSST start (T+0 minutes). **p* <.05 ***p* <.01 ****p* <.001 ^†^*p* <.10

**Supplemental Table 6. Primary participant sex two-way and three-way interaction models for log cortisol**

|  | Two-way: Condition x sex | Three-way: Condition x age group x sex |
| --- | --- | --- |
|  | ***b* (SE)** | ***b* (SE)** |
| Fixed effects |  |  |
| Intercept^a^ | 0.81 (0.12)*** | 0.66 (0.15)*** |
| Condition | -0.05 (0.15) | 0.26 (0.23) |
| Age group | 0.23 (0.11)* | 0.59 (0.17)*** |
| Sex | 0.35 (0.13)** | 0.58 (0.20)** |
| Time since wake | -0.03 (0.02) | -0.03 (0.02) |
| Medication | -0.09 (0.07) | -0.12 (0.07)^†^ |
| Condition x age group | -- | -0.64 (0.28)* |
| Condition x sex | 0.02 (0.21) | -0.38 (0.31) |
| Age group x sex | -- | -0.47 (0.26)^†^ |
| Condition x age x sex | -- | 0.84 (0.43)* |
| Linear slope | 0.66 (0.29)* | 0.87 (0.41)* |
| Condition | -0.07 (0.29) | -0.45 (0.47) |
| Age group | 0.08 (0.19) | -0.39 (0.46) |
| Sex | -0.21 (0.29) | -0.33 (0.48) |
| Time since wake | 0.05 (0.04) | 0.05 (0.05) |
| Medication | -0.15 (0.14) | -0.13 (0.13) |
| Condition x age group | -- | 0.80 (0.56) |
| Condition x sex | 0.11 (0.35) | 0.30 (0.57) |
| Age group x sex | -- | 0.32 (0.55) |
| Condition x age x sex | -- | 0.35 (0.71) |
| Quadratic growth | -0.91 (0.31)** | -1.02 (0.42)* |
| Condition | 0.18 (0.32) | 0.32 (0.50) |
| Age group | -0.26 (0.19) | -0.01 (0.51) |
| Sex | 0.17 (0.32) | 0.33 (0.51) |
| Time since wake | -0.10 (0.05)^†^ | -0.10 (0.05)^†^ |
| Medication | 0.16 (0.13) | 0.14 (0.13) |
| Condition x age group | -- | -0.30 (0.62) |
| Condition x sex | -0.16 (0.39) | -0.31 (0.61) |
| Age group x sex | -- | -0.32 (0.63) |
| Condition x age x sex | -- | 0.26 (0.78) |
| Random effects |  |  |
| Residual variance | 0.06 (0.01)*** | 0.06 (0.01)*** |
| Intercept | 0.36 (0.05)*** | 0.34 (0.04)*** |
| Linear slope | 0.75 (0.13)*** | 0.73 (0.13)*** |
| Quadratic growth | 0.52 (0.19)** | 0.52 (0.18)** |

Notes: Cortisol values were measured in nmol/L and natural log-transformed. Condition was coded as 0 = sibling and 1 = stranger. Age group was coded as 0 = child and 1 = adolescent. Sex was coded as 0 = female and 1 = male. Time since wake was mean-centered and medication was centered at zero. ^a^ Intercept represents log cortisol values for sibling condition at TSST start (T+0 minutes). **p* <.05 ***p* <.01 ****p* <.001 ^†^*p* <.10

**Supplemental Table 7. Sibling gender match two-way and three-way interaction models for log cortisol**

|  | Two-way: Condition x gender match | Three-way: Condition x age group x gender match |
| --- | --- | --- |
|  | ***b* (SE)** | ***b* (SE)** |
| Fixed effects |  |  |
| Intercept^a^ | 1.03 (0.11)*** | 1.05 (0.13)*** |
| Condition | -0.03 (0.15) | -0.08 (0.25) |
| Age group | 0.22 (0.12)^†^ | 0.18 (0.18) |
| Gender match | -0.01 (0.14) | -0.21 (0.22) |
| Time since wake | -0.04 (0.02)^†^ | -0.04 (0.03) |
| Medication | -0.14 (0.07)^†^ | -0.13 (0.07)* |
| Condition x age group | -- | 0.09 (0.31) |
| Condition x gender match | -0.08 (0.23) | 0.28 (0.34) |
| Age group x gender match | -- | 0.40 (0.28) |
| Condition x age x gender match | -- | -0.83 (0.46)^†^ |
| Linear slope | 0.50 (0.23)* | 0.47 (0.31) |
| Condition | 0.09 (0.25) | -0.15 (0.39) |
| Age group | 0.07 (0.19) | 0.15 (0.37) |
| Gender match | 0.11 (0.28) | 0.51 (0.49) |
| Time since wake | 0.05 (0.04) | 0.06 (0.05) |
| Medication | -0.13 (0.13) | -0.14 (0.13) |
| Condition x age group | -- | 0.42 (0.49) |
| Condition x gender match | -0.18 (0.37) | -0.37 (0.59) |
| Age group x gender match | -- | -0.79 (0.56) |
| Condition x age x gender match | -- | 0.46 (0.72) |
| Quadratic growth | -0.81 (0.22)*** | -0.63 (0.30)* |
| Condition | 0.04 (0.27) | 0.01 (0.44) |
| Age group | -0.24 (0.20) | -0.61 (0.35)^†^ |
| Gender match | -0.03 (0.30) | -0.50 (0.52) |
| Time since wake | -0.10 (0.05)* | -0.11 (0.05)* |
| Medication | 0.15 (0.13) | 0.16 (0.12) |
| Condition x age group | -- | 0.08 (0.53) |
| Condition x gender match | 0.07 (0.40) | 0.35 (0.64) |
| Age group x gender match | -- | 0.91 (0.62) |
| Condition x age x gender match | -- | -0.49 (0.78) |
| Random effects |  |  |
| Residual variance | 0.06 (0.01)*** | 0.06 (0.01)*** |
| Intercept | 0.39 (0.05)*** | 0.37 (0.05)*** |
| Linear slope | 0.78 (0.14)*** | 0.73 (0.12)*** |
| Quadratic growth | 0.54 (0.19)** | 0.52 (0.18)** |

Notes: Cortisol values were measured in nmol/L and natural log-transformed. Condition was coded as 0 = sibling and 1 = stranger. Age group was coded as 0 = child and 1 = adolescent. Gender match was coded as 0 = same gender and 1 = not the same gender. Time since wake was mean-centered and medication was centered at zero. ^a^ Intercept represents log cortisol values for sibling condition at TSST start (T+0 minutes). **p* <.05 ***p* <.01 ****p* <.001 ^†^*p* <.10

**Supplemental Table 8. Sibling Attachment Inventory (SAI) domain means by age group and depressive symptoms**

|  | | SAI subscale: Communication | SAI subscale: Trust | SAI subscale: Alienation |
| --- | --- | --- | --- | --- |
|  |  | **Mean (SE)** | **Mean (SE)** | **Mean (SE)** |
| Children  (9-11 years) | **Lower risk for depression** | 2.20 (0.07)^a^ | 2.70 (0.06)^a^ | 1.51 (0.05) |
|  | **Higher risk for depression** | 1.86 (0.80)^a,b^ | 2.31 (0.07)^a,b^ | 1.76 (0.05) |
| Adolescents  (15-17 years) | **Lower risk for depression** | 2.37 (0.08) | 2.80 (0.06) | 1.43 (0.05) |
|  | **Higher risk for depression** | 2.40 (0.08)^b^ | 2.84 (0.07)^b^ | 1.58 (0.06) |

Notes: Individuals who scored ≥15 on the CES-DC were classified as higher risk for depression.

^a^ Children at lower risk for depression reported significantly higher communication and trust than children at higher risk for depression ^b^ Adolescents at higher risk for depression reported significantly higher communication and trust than children at higher risk for depression.

**Supplemental Table 9. Condition x age group x depressive symptoms three-way interaction model for log cortisol, controlling for perceived helpfulness**

|  | *b* (SE) |
| --- | --- |
| Fixed effects |  |
| Intercept^a^ | 0.88 (0.13)*** |
| Condition | -0.20 (0.20) |
| Age group | 0.27 (0.16)^†^ |
| Sex | 0.33 (0.10)** |
| Time since wake | -0.03 (0.02) |
| Medication | -0.05 (0.06) |
| Depressive symptoms | -0.39 (0.20)^†^ |
| Condition x age group | 0.21 (0.29) |
| Condition x depressive | 0.67 (0.30)* |
| Age group x depressive | 0.15 (0.25) |
| Condition x age x depressive | -0.76 (0.41)^†^ |
| Perceived helpfulness | 0.11 (0.05)* |
| Linear slope | 0.36 (0.32) |
| Condition | 0.02 (0.36) |
| Age group | 0.26 (0.35) |
| Sex | -0.18 (0.19) |
| Time since wake | 0.04 (0.04) |
| Medication | -0.20 (0.15) |
| Depressive symptoms | 1.00 (0.48)* |
| Condition x age group | -0.03 (0.46) |
| Condition x depressive | -0.85 (0.58) |
| Age group x depressive | -1.24 (0.54)* |
| Condition x age x depressive | 1.60 (0.74)* |
| Intercept of log cortisol | 0.06 (0.14) |
| Perceived helpfulness | 0.03 (0.07) |
| Quadratic growth | -0.41 (0.37) |
| Condition | -0.23 (0.39) |
| Age group | -0.61 (0.34)^†^ |
| Sex | 0.22 (0.20) |
| Time since wake | -0.10 (0.05)* |
| Medication | 0.17 (0.15) |
| Depressive symptoms | -0.87 (0.54) |
| Condition x age group | 0.63 (0.48) |
| Condition x depressive | 1.02 (0.66) |
| Age group x depressive | 1.30 (0.63)* |
| Condition x age x depressive | -1.89 (0.84)* |
| Intercept of log cortisol | -0.25 (0.15)^†^ |
| Perceived helpfulness | 0.02 (0.08) |
| Random effects |  |
| Residual variance | 0.06 (0.01)*** |
| Intercept | 0.31 (0.04)*** |
| Linear slope | 0.67 (0.11)*** |
| Quadratic growth | 0.45 (0.16)** |

Notes: Cortisol values were measured in nmol/L and natural log-transformed. Condition was coded as 0 = sibling and 1 = stranger. Age group was coded as 0 = child and 1 = adolescent. Sex was coded as 0 = female and 1 = male. Depressive symptoms was coded as 0 = lower risk of depression and 1 = higher risk of depression. Time since wake and perceived helpfulness were mean-centered, and medication was centered at zero. ^a^ Intercept represents log cortisol values for sibling condition at TSST start (T+0 minutes). **p* <.05 ***p* <.01 ****p* <.001 ^†^*p* <.10

**Supplemental Table 10. Condition, age group, and depressive symptoms two-way and three-way interaction models for self-reported stress**

|  | Two-way: Condition x age group | Two-way: Condition x depressive symptoms | Three-way: Condition x age group x depressive symptoms |
| --- | --- | --- | --- |
|  | ***b* (SE)** | ***b* (SE)** | ***b* (SE)** |
| Fixed effects |  |  |  |
| Intercept^a^ | 2.40 (0.23)*** | 2.24 (0.22)*** | 2.06 (0.22)*** |
| Condition | -0.24 (0.27) | 0.05 (0.25) | 0.25 (0.32) |
| Age group | -0.20 (0.28) | -0.18 (0.19) | 0.20 (0.34) |
| Sex | -0.22 (0.20) | -0.20 (0.19) | -0.22 (0.19) |
| Depressive symptoms | -- | 0.34 (0.29) | 0.85 (0.41)* |
| Condition x age group | 0.06 (0.39) | -- | -0.40 (0.51) |
| Condition x depressive | -- | -0.57 (0.39) | -1.12 (0.55)* |
| Age group x depressive | -- | -- | -0.98 (0.57)^†^ |
| Condition x age x depressive | -- | -- | 1.07 (0.75) |
| Linear slope | 1.89 (0.19)*** | 1.92 (0.19)*** | 2.11 (0.17)*** |
| Condition | 0.21 (0.21) | 0.02 (0.22) | -0.03 (0.27) |
| Age group | -0.27 (0.23) | -0.25 (0.16) | -0.64 (0.28)* |
| Sex | 0.17 (0.17) | 0.16 (0.16) | 0.17 (0.16) |
| Depressive symptoms | -- | -0.09 (0.24) | -0.57 (0.32)^†^ |
| Condition x age group | 0.02 (0.33) | -- | 0.09 (0.44) |
| Condition x depressive | -- | 0.39 (0.33) | 0.59 (0.44) |
| Age group x depressive | -- | -- | 0.91 (0.47)^†^ |
| Condition x age x depressive | -- | -- | -0.28 (0.66) |
| Quadratic growth | -0.48 (0.05)*** | -0.49 (005)*** | -0.54 (0.04)*** |
| Condition | -0.04 (0.05) | -0.01 (0.05) | 0.00 (0.07) |
| Age group | 0.09 (0.06) | 0.07 (0.04)^†^ | 0.16 (0.07)* |
| Sex | -0.06 (0.04) | -0.06 (0.04) | -0.06 (0.04) |
| Depressive symptoms | -- | 0.05 (0.06) | 0.14 (0.08)^†^ |
| Condition x age group | -0.03 (0.08) | -- | -0.02 (0.11) |
| Condition x depressive | -- | -0.10 (0.08) | -0.12 (0.11) |
| Age group x depressive | -- | -- | -0.19 (0.11) |
| Condition x age x depressive | -- | -- | 0.02 (0.16) |
| Random effects |  |  |  |
| Residual variance | 0.90 (0.06)*** | 0.89 (0.06)*** | 0.88 (0.06)*** |
| Intercept | 0.55 (0.18)** | 0.54 (0.18)** | 0.53 (0.19)** |
| Linear slope | 0.01 (0.21) | 0.01 (0.21) | 0.01 (0.22) |
| Quadratic growth | 0.00 (0.01) | 0.00 (0.01) | 0.00 (0.01) |

Notes: Condition was coded as 0 = sibling and 1 = stranger. Age group was coded as 0 = child and 1 = adolescent. Sex was coded as 0 = female and 1 = male. Depressive symptoms was coded as 0 = lower risk of depression and 1 = higher risk of depression. ^a^ Intercept represents self-reported stress levels for sibling condition at arrival. *p* <.05 ***p* <.01 ****p* <.001 ^†^*p* <.10
